# Long-term safety and effectiveness of etanercept in JIA: an 18-year experience from the BiKeR registry

**DOI:** 10.1186/s13075-020-02326-5

**Published:** 2020-10-29

**Authors:** Giulia Armaroli, Ariane Klein, Gerd Ganser, Michael J. Ruehlmann, Frank Dressler, Anton Hospach, Kirsten Minden, Ralf Trauzeddel, Ivan Foeldvari, Jasmin Kuemmerle-Deschner, Frank Weller-Heinemann, Andreas Urban, Gerd Horneff

**Affiliations:** 1 Division of Paediatric Rheumatology, Sankt Augustin Asklepios Children’s Hospital, 53757 Sankt Augustin, Germany; 2 Cologne University, Medical School, Cologne, Germany; 3 Division of Paediatric Rheumatology, Northwest German Rheumatology Center, St. Josef Stift, Sendenhorst, Germany; 4 Paediatric Rheumatology Medical Center, Goettingen, Germany; 5 Division of Paediatric Pulmonology, Allergology and Immunology, Hannover Medical School, Hannover, Germany; 6 Division of Paediatric Rheumatology, Olgahospital, Stuttgart, Germany; 7 German Rheumatism Research Center, Charité University Hospital, Berlin, Germany; 8 Department of Paediatrics, Berlin-Buch Helios Hospital, Berlin, Germany; 9 Paediatric Rheumatology Medical Center, Hamburg, Germany; 10 Department of Paediatrics, Tuebingen University, Tuebingen, Germany; 11 Division of Paediatric Rheumatology, Prof. Hess Children’s Hospital, Bremen, Germany; 12 Department of Paediatrics, St. Marien Hospital, Amberg, Germany

**Keywords:** Juvenile Idiopathic Arthritis, JIA treatment, Etanercept, TNF inhibitors, Biologics registry, Drug surveillance

## Abstract

**Background:**

At present, etanercept represents the most commonly prescribed biologic agent for juvenile idiopathic arthritis (JIA) treatment. Children and adolescents with JIA are often treated with etanercept over long periods, sometimes even into adulthood. The objectives of this analysis were to determine the long-term safety of etanercept compared to a biologic-naïve cohort and to assess the long-term treatment response upon continuous etanercept exposure using data from the German biologics registry (BiKeR).

**Methods:**

JIA patients newly exposed to etanercept were documented in the BiKeR registry from January 2001 to March 2019, and baseline characteristics, effectiveness, and safety parameters were analysed. Response to treatment was assessed according to 10-joint Juvenile Arthritis Disease Activity Score (JADAS10), JADAS-defined minimal disease activity and remission, JIA-American College of Rheumatology (ACR) improvement criteria, and ACR-inactive disease definition. Safety assessments were based on adverse event (AE) reports.

**Results:**

A total of 2725 new etanercept users with a diagnosis of JIA were registered. Of these, etanercept was received as a first-line biologic by 95.8% and as monotherapy without concomitant methotrexate by 31.5%. After nine years on continuous treatment, 68.1% of patients presented minimal disease activity, 43.1% JADAS-defined remission on drug, and 36.6% ACR-inactive disease. JIA-ACR30/50/70/90 response rates were still 82/79/71/54% after nine years of treatment. Overall, 2053 AEs (34.3/100PY), including 226 serious AEs (SAE, 3.8/100PY), were observed upon etanercept, compared to 1345 AEs [35.6/100PY; *p* = 0.3] and 52 SAEs (1.4/100PY; *p* = 0.0001) in the biologic-naïve cohort. Respective exposure-adjusted rates for etanercept and biologic-naïve patients were 0.9/100PY and 0.2/100PY (*p* = 0.0001) for serious infections, 0.4/100PY and 0.1/100PY (*p* = 0.01) for zoster reactivation, 0.3/100PY and 0.03/100PY (*p* = 0.015) for inflammatory bowel disease, and 1.9/100PY and 1.4/100PY (*p* = 0.09) for uveitis. Three and two malignancies were documented in the etanercept and biologic-naïve groups, as well as three and one deaths, respectively.

**Conclusions:**

No new safety signal was observed, especially no increased risk for malignancies or autoimmune disorders other than inflammatory bowel disease. However, SAEs and serious infections, though infrequent, were more often reported on etanercept than in biologic-naïve patients. In addition, etanercept demonstrated a long-term maintenance of clinical benefits up to nine years of continuous treatment.

## Background

Treatment of juvenile idiopathic arthritis (JIA) represents a major challenge in paediatric rheumatology. Diverse treatment options are currently available. Methotrexate is the most commonly prescribed conventional disease-modifying anti-rheumatic drug (DMARD). Within biologics, etanercept, a tumour necrosis factor inhibitor (TNFi), was the first drug to be approved for JIA in 2001 and represents at present the favoured first-line biologic agent for JIA patients [1]. Etanercept is approved for use in polyarticular JIA in children older than 2 years of age and for use in psoriatic arthritis (PsA) and enthesitis-related arthritis (ERA) in patients older than 12 years of age. For systemic JIA or persistent oligoarthritis, etanercept is not approved, so that its use in these conditions is mostly reserved for children who experience refractory disease [2]. From 2001 to present, the increasing use of etanercept in patients with JIA has raised awareness of rare serious adverse events, such as malignancies and autoimmune conditions, including, but not limited to, uveitis, inflammatory bowel disease, and demyelinating disorders [3–5]. Also, etanercept treatment may be required over many years in JIA patients, sometimes even into adulthood. Yet, knowledge about its safety and effectiveness in the long-term is limited.

The German registry for biologics in paediatric rheumatology (BiKeR) is one of the largest national registries on the use of biologics in JIA. Over a period of 18 years, it has accumulated a large quantity of data on etanercept-treated JIA patients. We performed a systematic review of the BiKeR registry to evaluate the long-time safety and effectiveness of etanercept in JIA. A biologic-naïve cohort was used as a comparator for long-term safety analyses.

## Methods

The German BiKeR registry has been documenting treatment of JIA with biologics since 2001 and has been extensively described in previous reports [6, 7]. It was approved by the ethics committee of the physician board Aerztekammer Nordrhein, Duesseldorf. The BiKeR registry is registered in the European Network of Centres for Pharmacoepidemiology and Pharmacovigilance (ENCePP [8]). Written consent was obtained from patients and parents, and repeated when the patient became an adult. Pseudonymized data were collected for each JIA patient starting a biologic therapy and belonging to the seven ILAR-defined JIA categories [9] as determined by the reporting physician. Dose and frequency of administration were documented. Patient assessment regarding effectiveness and occurrence of adverse events (AEs) was performed at baseline and at follow-up after three and six months and every six months thereafter. After discontinuation of treatment, patients were followed up every six months with a request to report any AE, and patients transitioning to adult care are followed up by the JuMBO registry [10]. Patients of the registry newly starting treatment with etanercept from January 2001 to March 2019 were included in the study if they had assessments at baseline and at least at the three-month visit, irrespective of diagnosis. All follow-up forms received prior to April 2019 were evaluated. Reasons for discontinuation of etanercept treatment were also documented. Multiple reasons could be given. JIA patients who newly started methotrexate treatment and never received biologics were recruited between 2005 and 2011 till inclusion of 1500 patients and served as the control group for long-term safety analyses.

### Assessment of effectiveness

Effectiveness parameters were defined as follows. The JIA-American College of Rheumatology (ACR) improvement criteria and the Juvenile Arthritis Disease Activity Score (JADAS) were calculated as previously described [11, 12]. JIA-ACR core set parameters consist of (i) physician global assessment of disease activity (PhysVAS) on a 10-cm visual analogue scale (VAS); (ii) parent/patient global assessment of overall well-being (PatVAS) on a 10-cm VAS; (iii) the Childhood Health Assessment Questionnaire (CHAQ); (iv) the number of joints with active arthritis, defined by the presence of swelling or, if no swelling is present, limitation of motion accompanied by pain, tenderness, or both; (v) the number of joints with limited range of motion; and (vi) the erythrocyte sedimentation rate (ESR). The ACR-inactive disease definition was used according to Wallace et al. [13], requiring no active uveitis or arthritis, no fever, rash, splenomegaly, serositis, generalised lymphadenopathy or elevation of ESR/C-reactive protein (CRP), best possible PhysVAS, and duration of morning stiffness ≤ 15 min. JADAS10 was chosen, which considers a maximum of ten active joints besides PatVAS, PhysVAS, and ESR or CRP, all equally weighted. Rates of JADAS-minimal disease activity (MDA) and JADAS-remission, respectively defined as JADAS10 ≤ 3.8 and JADAS10 ≤ 1, were calculated according to the definition of Consolaro et al. [14]. For each timepoint, all patients with complete data set were considered. Data from patients who discontinued etanercept treatment due to remission were analysed for disease activity also after therapy withdrawal, and rates of JADAS-MDA and JADAS-remission off-biologics were determined.

### Safety analysis

Safety was analysed based on AE reporting for all patients during the whole treatment period. AEs and serious AEs (SAEs) were defined according to the International Council for Harmonisation of Technical Requirements for Pharmaceuticals for Human Use (ICH) E6 Section 1.2 [15]. Exposure-adjusted AE rates were calculated per 100 patient-years (PY) with 95% confidence interval (CI). AEs and SAEs were attributed to the etanercept treatment if the patient had been treated with etanercept at the time of the occurrence of the AE or during the last 90 days prior to the AE occurrence, regardless of a possible cotreatment with methotrexate. Malignancies, pregnancies, and deaths were additionally analysed in the ever-treated population.

### Statistical analysis

For the comparison of baseline characteristics, the chi-squared, Fisher’s exact, or Mann-Whitney *U* test was used, depending on data distribution. Mean changes from baseline in each effectiveness parameter were compared using the unpaired *t* test. Differences in AE rates were analysed using risk ratios (RRs) and the Wald test. A *p* value < 0.05 was considered statistically significant. Analyses were conducted with IBM SPSS Statistics version 23 (IBM Corp., Armonk, NY, USA) and SAS 9.3 (SAS Institute, Cary, NC, USA).

## Results

### Study population

**Fig. 1:**
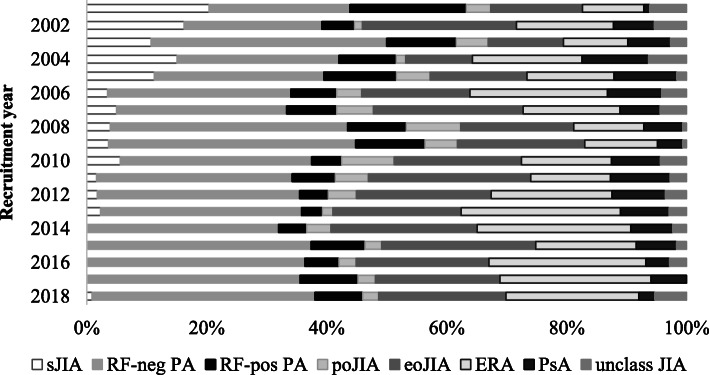
Subtype distribution per recruitment year in the etanercept cohort. sJIA, systemic juvenile idiopathic arthritis; RF-neg PA, rheumatoid factor-negative polyarthritis; RF-pos PA, rheumatoid factor-positive polyarthritis; poJIA, persistent oligoarthritis; eoJIA, extended oligoarthritis; ERA, enthesitis-related arthritis; PsA, psoriatic arthritis; unclass JIA, unclassified juvenile idiopathic arthritis

Within the JIA patients who initiated etanercept treatment for the first time between 2001 and 2019, 2725 were eligible for the analysis. Mean etanercept dose was 0.79 ± 0.21/kg/week. The administration regimen was once weekly in 56.9% of patients and twice weekly in 43.1%. Overall, the majority of etanercept-treated patients in Germany showed to be diagnosed with (rheumatoid factor (RF)-negative) polyarthritis, extended oligoarthritis, or ERA, although the cohort subtype distribution varied considerably over the years (Fig. 1). Rates of etanercept-treated patients with RF-negative polyarthritis and with ERA increased over time (23.5 and 9.8% in 2001, 37.2 and 22.1% in 2018, respectively), while percentages of patients with systemic JIA or RF-positive polyarthritis decreased (20.4 and 19.4% in 2001, 0.9 and 8.0% in 2018, respectively). The comparison cohort of 1517 JIA biologic-naïve patients starting methotrexate significantly differed from the etanercept cohort for subtype distribution (Table 1). Age at disease onset was comparable between the cohorts, while age at baseline was significantly higher in the etanercept group (12.1 ± 4.4 versus 9.8 ± 4.8 years; *p* < 0.0001; Table 1). Disease duration at baseline, as calculated from symptom onset to start of cohort defining treatment, was also significantly higher in the etanercept cohort (4.1 ± 3.7 versus 2.1 ± 2.8 years; *p* < 0.0001). However, a significant decrease in the mean disease duration at etanercept start was observed over time (from 6.0 ± 3.9 in 2001 to 3.3 ± 3.1 years in 2018; *p* = 0.0001; Supplementary figure S1a). At baseline, 86.5% of etanercept patients had been pretreated with methotrexate, while only 4.2% had been preexposed to other biologics. Concomitant treatment at baseline with systemic steroids was observed more frequently in the biologic-naïve group (91.9 versus 35.7%; *p* = 0.0001; Table 1). All disease activity parameters at baseline, except for mean ESR levels, were significantly higher in the etanercept cohort. However, the mean JADAS10 at etanercept treatment initiation showed to decrease significantly over the years (from 20.6 ± 7.8 in 2001 to 10.7 ± 5.8 in 2018; *p* = 0.003; Supplementary figure S1b).

**Table 1:** Baseline characteristics

	Etanercept cohort (*N* = 2725)	Biologic-naïve cohort (*N* = 1517)	*p*^†^
Gender, female	1829 (67.1)	1023 (67.4)	0.8
Age at onset (years)	7.9 ± 4.7	7.7 ± 4.6	0.18
Age at baseline (years)	12.1 ± 4.4	9.8 ± 4.8	< 0.0001*
Disease duration (years)	4.1 ± 3.7	2.1 ± 2.8	< 0.0001*
JIA category			
Systemic JIA	146 (5.3)	58 (3.8)	0.025*
RF-negative polyarthritis	904 (33.1)	415 (27.3)	< 0.0001*
RF-positive polyarthritis	223 (8.1)	52 (3.4)	< 0.0001*
Persistent oligoarthritis	120 (4.4)	390 (25.7)	< 0.0001*
Extended oligoarthritis	570 (20.9)	204 (13.4)	< 0.0001*
ERA	486 (17.8)	213 (14.0)	0.001*
PsA	191 (7.0)	138 (9.0)	0.017*
Unclassified JIA	85 (3.1)	47 (3.0)	1.0
ANA	1290 (47.3)	725 (47.8)	0.8
HLA-B27	643 (23.6)	265 (17.5)	< 0.0001*
Pretreatment at baseline			
NSAIDs	2478 (90.9)	1329 (87.6)	0.0007*
Systemic steroids	1434 (52.6)	357 (23.5)	< 0.0001*
MTX	2358 (86.5)	0 (0)	< 0.0001*
Biologics	114 (4.2)	0 (0)	< 0.0001*
Other DMARDs	1232 (45.2)	149 (9.8)	< 0.0001*
SFZ	415 (15.2)	67 (4.4)	< 0.0001*
HCQ	228 (8.4)	53 (3.5)	< 0.0001*
AZA	237 (8.7)	14 (0.9)	< 0.0001*
LEF	85 (3.1)	6 (0.4)	< 0.0001*
CSA	140 (5.1)	8 (0.5)	< 0.0001*
Chlorambucil	19 (0.7)	0 (0)	0.0004*
Cyclophosphamide	10 (0.4)	0 (0)	0.0175*
Gold salts	36 (1.3)	0 (0)	< 0.0001*
Immunoglobulins	48 (1.8)	1 (0.1)	< 0.0001*
MMF	14 (0.5)	0 (0)	0.0034*
Concomitant treatment at baseline			
NSAIDs	2158 (79.2)	360 (23.7)	0.0001*
Systemic steroids	974 (35.7)	1394 (91.9)	0.0001*
MTX	1867 (68.5)	1517 (100.0)	< 0.0001*
Other DMARDs			
SFZ	145 (5.3)	36 (2.4)	0.0001*
HCQ	37 (1.4)	14 (0.9)	0.24
AZA	80 (2.9)	4 (0.3)	0.0001*
LEF	59 (2.2)	1 (0.1)	0.0001*
CSA	57 (2.1)	2 (0.1)	0.0001*
Disease activity parameters at baseline			
Active joints	6.7 ± 8.1	5.8 ± 7.6	0.0004*
Swollen joints	5.3 ± 7.4	4.8 ± 6.8	0.03*
Tender joints	6.5 ± 8.4	5.8 ± 7.8	0.007*
PhysVAS	52.2 ± 32.3	47.2 ± 25.9	0.0001*
PatVAS	43.7 ± 27.4	39.0 ± 26.0	0.0001*
Joints with LOM	7.4 ± 8.9	5.7 ± 7.6	0.0001*
CHAQ-DI	0.7 ± 0.6	0.6 ± 0.6	0.0001*
ESR (mm/h)	23.5 ± 23.4	24.2 ± 23.0	0.35
CRP (mg/L)	16.8 ± 32.7	13.9 ± 27.9	0.004*
JADAS10	15.3 ± 7.5	13.9 ± 7.1	0.0001*

Data are shown as *n* (%), mean ± SD, or *n*

*JIA* juvenile idiopathic arthritis, *RF* rheumatoid factor, *ERA* enthesitis-related arthritis, *PsA* psoriatic arthritis, *ANAs* antinuclear antibodies, *HLA* human leucocyte antigen, *NSAID* nonsteroidal anti-inflammatory drug, *MTX* methotrexate, *DMARD* disease-modifying anti-rheumatic drug, *SFZ* sulfasalazine, *HCQ* hydroxychloroquine, *AZA* azathioprine, *LEF* leflunomide, *CSA* cyclosporine, *PhysVAS* physician global assessment of overall well-being, *PatVAS* parent/patient global assessment of overall well-being, *LOM* limitation of motion, *CHAQ*-DI Childhood Health Assessment Questionnaire disability index, *ESR* erythrocyte sedimentation rate, *CRP* C-reactive protein, *JADAS10* 10-joint Juvenile Arthritis Disease Activity Score

^†^By *t* test or Fisher’s exact test, as appropriate

*p < 0.05

### Effectiveness

**Fig. 2:**
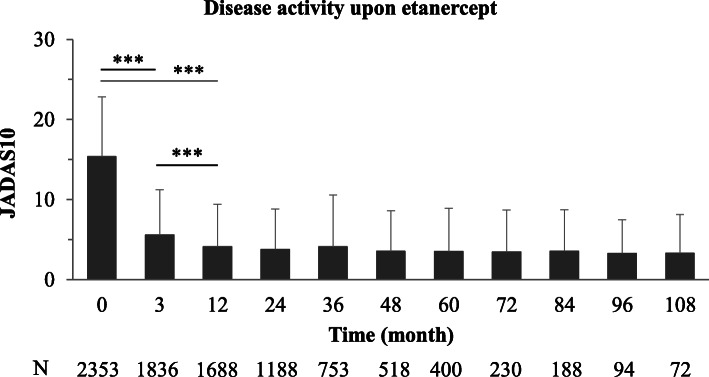
Mean JADAS10 as efficacy measure over time. Mean 10-joint Juvenile Arthritis Disease Activity Score (JADAS10) in JIA patients over the course of etanercept treatment. ***p < 0.001

On etanercept, the mean JADAS10 decreased from 15.3 ± 7.5 at baseline to 5.6 ± 5.7 (*p* < 0.0001) after 3 months and to 4.1 ± 5.3 (*p* < 0.0001) after 12 months of treatment (Fig. 2). Patients recruited in the most recent years achieved a lower JADAS10 after 12 months on etanercept compared to those enrolled in the earlier years, although the difference did not reach significance (2.3 ± 2.0 in 2018 compared to 6.9 ± 7.2 in 2001; *p* = 0.0637; Supplementary figure S1b).

**Fig. 3:**
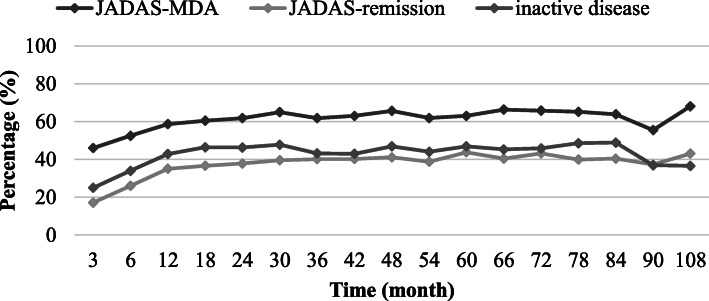
Measures of treatment efficacy over time. Rates of Juvenile Disease Activity Score (JADAS)10-minimal disease activity (JADAS10 ≤ 3.8) and JADAS10-remission (JADAS10 ≤ 1) and rates of patients reaching inactive disease according to Wallace et al. [13] upon etanercept treatment

JADAS-defined minimal disease activity (MDA; JADAS ≤ 3.8) was reached at months 3, 12, and 24 in 844 (45.9%), 990 (58.6%), and 734 (61.8%) etanercept patients, and in 252 (63.0%), 120 (63.8%), and 49 (68.1%) patients after 5, 7, and 9 years. JADAS-remission (JADAS ≤ 1) was reached at months 3, 12, and 24 in 315 (17.2%), 591 (35.0%), and 449 (37.8%) patients, and in 175 (43.8%), 76 (40.4%), and 31 (43.1%) patients after 5, 7, and 9 years (Fig. 3). Over the course of the years, the percentage of patients who reached JADAS-MDA and JADAS-remission following 12 months of etanercept treatment increased, respectively, from 43.1 and 20.9% in 2001 to 72.8 and 45.6% in 2018 (Supplementary figure S1c).

**Fig. 4:**
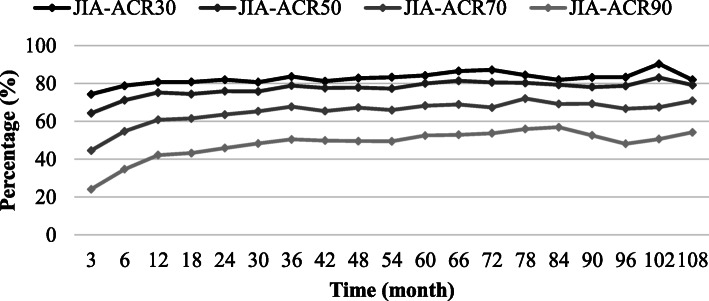
ACR improvement rates over time. Improvement rates of disease activity parameters over the course of etanercept treatment according to the JIA-American College of Rheumatology (ACR) criteria

ACR-inactive disease according to Wallace et al. [13] was reached at months 3, 12, and 24 by 166 (25.0%), 248 (42.8%), and 180 (46.3%) etanercept patients, and in 84 (46.9%), 43 (48.9%), and 15 (36.6%) patients after 5, 7, and 9 years (Fig. 3). Improvement according to JIA-ACR30/50/70/90 criteria was reached in 74/64/45/24% of patients at month 3, in 81/75/61/42% of patients at month 12, and in 82/76/64/46% of patients at month 24 (Fig. 4). JIA-ACR30/50/70/90 response rates were 84/80/68/53% after 5 years, 82/79/69/57% after 7 years, and 82/79/71/54% after 9 years on etanercept.

The effectiveness parameters JADAS-MDA, JADAS-remission, and ACR-inactive disease were also evaluated in an intention-to-treat (ITT) analysis, with patients who discontinued due to remission considered as responders, and patients discontinuing for any other reason as non-responders. In this analysis, JADAS-MDA was reached by 40.2, 41.2, and 33.8% of patients at months 3, 12, and 24, and by 21.3, 19.7, and 19.0% after 5, 7, and 9 years. JADAS-remission was reached by 15.1, 25.6, and 23.9% of patients at months 3, 12, and 24, and by 19.7, 19.1, and 18.7% after 5, 7, and 9 years. ACR-inactive disease was reached by 18.8, 27.0, and 25.0% of patients at months 3, 12, and 24, and by 19.6, 19.2, and 18.8% after 5, 7, and 9 years.

### Discontinuations and remission rates off-biologics

Over 18 years of observation, etanercept was discontinued by 1655 (60.7%) patients. Rates and reasons for discontinuation are listed in Table 2. The most common reason for discontinuation was remission (23.9%), followed by inefficacy (21.8%) and intolerance (7.1%). Of the 652 patients discontinuing due to remission, 521 patients had available data one year after etanercept withdrawal. In the first year, 126 (24%) patients relapsed, and 395 (76%) remained without etanercept or other biologic agents. Of these, 214 (54%) maintained at least JADAS-MDA and 121 (31%) remained in JADAS-remission off-biologics (Supplementary figure S2). Five years after etanercept discontinuation, 105 patients remained without etanercept or other biologic agents, of which 27 (26%) maintained at least JADAS-MDA and 11 (11%) JADAS-remission off-biologics.

**Table 2:** Rates and reasons for discontinuation

	Etanercept cohort (*N* = 2725)
Discontinuations	1655 (60.7)
Remission	652 (23.9)
Inefficacy	594 (21.8)
Intolerance	192 (7.1)
Patient’s demand	504 (18.5)
Others	287 (10.5)

Data are shown as *n* (%). Multiple reasons could be given

### Safety

During 5988 patient-years of etanercept exposure, a total of 2053 AEs were reported to the registry (Table 3). No significant difference in exposure-adjusted AE rates was observed between etanercept (34.3/100PY) and biologic-naïve patients (35.6/100PY; *p* = 0.3). The AEs qualifying as serious (SAEs) were significantly more frequent in the etanercept cohort (3.8 versus 1.4/100PY; *p* = 0.0001). The incidence of serious infections was significantly higher in the etanercept group (0.9 versus 0.2/100PY; *p* = 0.0001), while neutropenia rates were comparable in the two cohorts (0.07 versus 0.05/100PY; *p* = 0.8). All reported opportunistic infections, but for one case of latent tuberculosis, were herpes zoster reactivation, and were more often observed under etanercept (0.4 versus 0.1/100PY; *p* = 0.01). The case of latent tuberculosis consisted in a positive Quantiferon Gold test without any clinical symptom or change in chest radiograph and was documented in a patient on methotrexate and with previous etanercept treatment.

**Table 3:** Safety assessment: adverse event (AE) reports

	Etanercept, 5988PY	Biologic-naïve, 3782PY	RR (95% CI)	*p*^†^
	E/E/100PY (95% CI)	E/E/100PY (95% CI)		
AE	2053/34.3 (32.8–35.8)	1345/35.6 (33.7–37.5)	1.0 (0.9–1.03)	0.3
SAE	226/3.8 (3.3–4.3)	52/1.4 (1.1–1.8)	2.8 (2.0–3.7)	0.0001*
Serious infection	54/0.9 (0.7–1.2)	8/0.2 (0.1–0.4)	4.3 (2.0–9.0)	0.0001*
Herpes zoster	24/0.4 (0.3–0.6)	4/0.1 (0.04–0.3)	3.8 (1.3–10.9)	0.01*
Neutropenia	4/0.07 (0.03–0.18)	2/0.05 (0.01–0.2)	1.3 (0.2–6.9)	0.8
MAS	2/0.03 (0.008–0.3)	1/0.03 (0.004–0.19)	1.3 (0.1–13.9)	0.9
High transaminases	97/1.6 (1.3–2.0)	175/4.6 (4.0–5.4)	0.4 (0.3–0.5)	0.0001*
IBD	19/0.3 (0.2–0.5)	1/0.03 (0.004–0.19)	12.0 (1.6–89.7)	0.015*
Uveitis	113/1.9 (1.6–2.3)	54/1.4 (1.1–1.9)	1.3 (0.96–1.8)	0.09
Psoriasis	4/0.07 (0.03–0.18)	1/0.03 (0.004–0.19)	2.5 (0.3–22.6)	0.4
Demyelination	1/0.02 (0.002–0.1)	0/n.a.	n.a.	n.a.
Depression	15/0.25 (0.2–0.4)	2/0.05 (0.01–0.2)	4.7 (1.1–20.7)	0.04*
Malignancy	3/0.05 (0.02–0.2)	2/0.05 (0.01–0.2)	1.0 (0.2–5.7)	0.95
Death	3/0.05 (0.02–0.2)	1/ 0.03 (0.004–0.19)	1.9 (0.2–18.2)	0.6

*PY* patient-years, *E* event, *E/100PY* rate, *CI* confidence interval, *RR* risk ratio, *SAE* serious adverse event, *MAS* macrophage activation syndrome, *IBD* inflammatory bowel disease, *n.a.* not applicable

^†^By Wald’s test

*p < 0.05

Inflammatory bowel disease (IBD) occurred with significantly greater frequency in etanercept patients (0.3 versus 0.03/100PY; *p* = 0.015). Nineteen patients developed IBD on etanercept (14 female, 3 HLA-B27 positive). Of these, six patients were diagnosed with RF-negative polyarthritis, six with extended polyarthritis, four with ERA (of which three male and two HLA-B27 positive), and one each with RF-positive polyarthritis, PsA, and systemic JIA. Two developed a sacroiliitis, one an iridocyclitis, and one an alpha-1-antitrypsin deficiency. Mean age at arthritis onset was 6.8 ± 4.3 years, and mean age at IBD onset was 13.7 ± 2.7 years. Patients developed IBD after 2.3 ± 1.9 years on ongoing etanercept therapy. All 19 patients had been pretreated with MTX. Rates of psoriasis (0.07 versus 0.003/100PY; *p* = 0.4) and aggravation/new onset of uveitis (1.9 versus 1.4/100PY; *p* = 0.09) did not differ significantly between the two analysed cohorts. One patient in the etanercept cohort developed demyelination and none in the biologic-naïve control cohort. The lesion, a minor alteration of the periventricular white matter, was discovered incidentally in an asymptomatic patient and has been described earlier [7]. Other reported immune-mediated events were Henoch-Schonlein purpura, leukocytoclastic cutaneous vasculitis, and lupus-like syndrome in one patient each. All three patients were on etanercept treatment.

Fifteen reports of suicidal intention or ideation, suicide attempt, or depression were documented in the etanercept cohort. Six were observed in RF-negative and one in RF-positive polyarthritis patients, two cases in extended and one in persistent oligoarthritis patients, and two in ERA and three in PsA patients. In all, the occurrence of suicide intention/depression was significantly higher in the etanercept group than in the biologic-naïve group (0.25 versus 0.05/100PY; *p* = 0.04).

Three pregnancies occurred in patients under etanercept treatment at the time of conception. A 17-year-old patient who was treated with etanercept and methotrexate delivered at term a healthy 3360-g male infant after a pregnancy without complications. Treatment was interrupted as her pregnancy was diagnosed at 6 weeks of gestation. The child was developing normally at two months of age. An 18-year-old patient gave birth to a healthy male infant, weight and gestational age of which have not been reported by the documenting physician. At the age of six months, the child showed normal growth and development. The third patient decided on an induced abortion at 12 weeks of gestation. Additionally, a miscarriage after 12 weeks of gestation was reported to the registry in an 18-year-old patient, two years after discontinuation of etanercept. She had been treated with etanercept 50 mg weekly over nine months. The patient received hydroxychloroquine 300 mg daily from 20 months before conception through eight weeks of pregnancy. No pregnancy was recorded in the biologic-naïve cohort.

Three malignancies were documented in patients on etanercept at the time of diagnosis (0.05/100PY). An 18-year-old male patient developed a non-familial thyroid carcinoma. One case each of Hodgkin’s and non-Hodgkin’s lymphoma was reported in two male patients. Malignancies were reported to the registry in five other patients who had been exposed to etanercept in the past: two cases of lymphoproliferative disorder and one case each of anaplastic ependymoma, yolk sac carcinoma, and cervix dysplasia. All patients recovered. In the biologic-naïve cohort with methotrexate, two cases of acute lymphatic leukaemia (ALL) were documented (0.05/100PY). One patient recovered; the second died. All malignancy cases have been previously described [16].

In all patients ever treated with etanercept, five deaths were reported, three of these during drug exposure. Two deaths occurred during adolescence and three in adulthood. One patient with systemic JIA died due to septic shock while on treatment with etanercept, after having been pretreated with cyclophosphamide and chlorambucil years before. A second patient with systemic JIA succumbed to heart failure by macrophage activation syndrome (MAS), one year after discontinuation of etanercept due to inefficacy. Both deaths occurred at the age of 16 years and have been formerly reported [17]. Of the three deaths during adulthood, one occurred in a 22-year-old due to perimyocarditis with arrhythmia, eight weeks after voluntary discontinuation of etanercept. A second patient died at the age of 22 years by suicide, seven years after etanercept discontinuation, and a third one died at the age of 23 years due to pseudomembranous enterocolitis by a septic urinary tract infection with renal failure and pancytopenia after 13 years of etanercept exposure. The events were considered as not related to etanercept treatment. In the biologic-naïve group, one death was reported. A 13-year-old female patient on methotrexate succumbed to ALL.

## Discussion

The current registry study represents the largest cohort of etanercept-treated JIA patients studied. To our knowledge, it is the first report on safety and effectiveness of etanercept including all JIA categories and following patients up to nine years of continuous treatment.

A significant improvement from baseline was observed in all analysed efficacy parameters already after three months of treatment. These improvements were maintained during up to nine years of sustained drug use, in accordance to observations from previous studies [18–20]. Patients who had been recruited in most recent years (2016–2018) had higher JADAS-MDA and JADAS-remission responses after one year of treatment compared with patients recruited in earlier years (2001–2003). This is likely reflective of the shorter disease duration and lower disease activity at the start of treatment of the formers, supporting the increasing evidence in the literature indicating a positive prognostic effect of an early aggressive treatment due to a suggested window of opportunity [21].

Next to an as-observed analysis evaluating disease activity in patients who continued treatment, we performed an effectiveness analysis in the ITT population. In this analysis, the percentage of patients reaching JADAS-remission or ACR-inactive disease stayed stable over time, while the percentage of patients presenting JADAS-MDA decreased over time. However, this represents a conservative assessment, since it presumes that all patients lost to follow-up did poorly, while only 28.9% of the patients discontinued due to inefficacy or intolerance. Of note, one third of the patients who discontinued due to remission remained in clinical remission off-biologics one year after etanercept withdrawal.

Incidence of adverse events was low, and no new safety signal emerged with long-term continuous etanercept exposure. Rates of SAE and serious infection were higher than in biologic-naïve patients, remaining yet low in both groups. By interpreting these data, it should be considered that patients initiating etanercept, according to JIA therapy recommendations, were mostly those who failed to respond or responded inadequately to treatment with ≥ 1 conventional DMARD, hence, those with a refractory JIA. They presented longer disease duration and higher disease activity, and had received more previous treatments, so one or more of these factors may have accounted for the higher serious infection rate. In fact, as described by Beukelman et al. [22], it is hypothesised that, in JIA patients, the underlying disease process itself, independently from treatment, might increase the risk of serious infections. The comedication with methotrexate, documented in two thirds of the etanercept cohort, may be also a contributing factor, as it was showed in prior studies that serious infections had a higher incidence in patients treated with etanercept-methotrexate combination treatment compared to monotherapy [3]. The rates for serious infections in the here analysed etanercept cohort were similar to those reported in other long-term registry studies [19, 20], but lower than what described in long-term controlled clinical trials [18, 23]. While other studies have also found higher serious infection rates upon etanercept [3, 24], others have described comparable rates to non-biological treatments. A large study comparing Medicaid data from 2713 new TNFi users found no significant difference in hospitalised infection rates between TNFi and methotrexate treatments [25]. In our study, herpes zoster reactivation was the only opportunistic infection reported, and it was more often observed in etanercept than in biologic-naïve patients with methotrexate, similarly to what described by other groups [23, 24].

Incidence of new-onset IBD was higher in etanercept than in biologic-naïve patients, which is consistent with previous findings [26]. Since etanercept was shown to be ineffective in Crohn’s disease, gastrointestinal manifestations in patients with IBD-associated arthritis may occur more likely under treatment with etanercept [27]. Rates of other autoimmune disorders, mostly psoriasis and uveitis, were comparable within the two groups. Only one case of suspected demyelination has been reported to our registry in conjunction with etanercept exposure. In the data from the large multinational Pharmachild/PRINTO pharmacovigilance study, demyelination was also a rare event [28].

Prior studies have suggested that patients with JIA may be at increased risk of malignancy [4]. In our study, treatment with etanercept did not associate with a higher incidence of malignancies. Similar results were reported in a Scandinavian registry study [5] as well as in the Pharmachild registry [28].

The interpretation of the results provided here is potentially influenced by the classic limitations accompanying registry studies, such as lack of blinding, lack of randomisation, and, for the long-term efficacy analysis, lack of an internal comparator. Comparison analyses between the two groups in terms of efficacy were, for the nature itself of the study as a registry analysis and not a randomised controlled clinical trial, not feasible. In addition, the majority of the etanercept patients had already received methotrexate and, as per JIA treatment recommendations, had failed to respond or responded inadequately to it, if a switch or escalation of treatment was required. Moreover, in two thirds of etanercept patients, methotrexate was not discontinued, but etanercept was added to it. Similarly, the biologic-naïve group used as a comparator for the long-term safety assessment presented significant differences at baseline to the etanercept group. This is due to the fact that while the latter had a refractory and more active disease, the former had just begun treatment with methotrexate as first-line DMARD.

Yet, registry studies remain of great importance because they reflect routine care and allow investigation of safety and effectiveness in a complete spectrum of patients and in a real-world setting. Differently, decisions in randomised clinical trials (RCTs) may be influenced by protocol and inclusion/exclusion criteria generate a mostly homogeneous study population of selected patients, e.g. by excluding determinate subtypes, comorbidities, or concomitant drugs. Moreover, in the present analysis, the high number of patients and the long study period allow detection of rare adverse events and adverse events occurring with long-term exposure.

## Conclusions

While no increased rates of malignancies and autoimmune disorder other than IBD were observed under etanercept, SAE and serious infection ratios were lower in the biologic-naïve patients with methotrexate, highlighting the high tolerability of the latter. Moreover, long-term etanercept treatment demonstrated a sustained efficacy in this large cohort of JIA patients.

## Supplementary information

**Additional file 1: Supplementary figure S1.** Disease duration and activity per recruitment year in the etanercept cohort. (a) Disease duration at the start of etanercept treatment per recruitment year. (b) Mean 10-joint Juvenile Arthritis Disease Activity Score (JADAS10) at baseline and after 12 months of etanercept treatment per recruitment year. (c) Patients reaching JADAS-minimal disease activity (MDA) and JADAS-remission at month 12 upon etanercept per recruitment year. ***p < 0.001, **p < 0.01.

**Additional file 2: Supplementary figure S2.** Clinical remission during eight years of follow-up following etanercept withdrawal. Rates of JADAS-minimal disease activity (MDA) and JADAS-remission off-biologics in patients who discontinued etanercept after achieving a stable clinical remission.

## Data Availability

Not applicable.

## Abbreviations

ACR: American College of Rheumatology
AE: Adverse event
ALL: Acute lymphatic leukaemia
BiKeR: Biologics in paediatric rheumatology registry
CHAQ: Childhood Health Assessment Questionnaire
CI: Confidence interval
CRP: C-reactive protein
DMARD: Disease-modifying anti-rheumatic drug
ENCePP: European Network of Centres for Pharmacoepidemiology and Pharmacovigilance
ERA: Enthesitis-related arthritis
ESR: Erythrocyte sedimentation rate
HLA: Human leucocyte antigen
IBD: Inflammatory bowel disease
ICH: International Council for Harmonisation of Technical Requirements for Pharmaceuticals for Human Use
IL: Interleukin
ILAR: International League of Associations for Rheumatology
JADAS: Juvenile Disease Activity Score
JADAS10: 10-joint Juvenile Disease Activity Score
JIA: Juvenile idiopathic arthritis
JIA-ACR: American College of Rheumatology improvement criteria for JIA
JRA: Juvenile rheumatoid arthritis
JuMBO: Juvenile arthritis-methotrexate-biologics long-term observation
MAS: Macrophage activation syndrome
MDA: Minimal disease activity
NSAIDs: Nonsteroidal anti-inflammatory drugs
PatVAS: Parent/patient global assessment of overall well-being on a 10-cm VAS
PhysVAS: Physician global assessment of disease activity on a 10-cm VAS
PRINTO: Paediatric Rheumatology International Trials Organisation
PsA: Psoriasis arthritis
PY: Patient-years
RCTs: Randomised clinical trials
RF: Rheumatoid factor
RR: Risk ratio
SAE: Serious adverse event
SD: Standard deviation
TNF: Tumour necrosis factor
TNFi: Tumour necrosis factor inhibitor
VAS: Visual analogue scale

## References

[1] Ravelli A, Consolaro A, Horneff G, Laxer RM, Lovell DJ, Wulffraat NM (2018). Treating juvenile idiopathic arthritis to target: recommendations of an international task force. Ann Rheum Dis.

[2] Ringold S, Weiss PF, Beukelman T, Dewitt EM, Ilowite NT, Kimura Y (2013). 2013 update of the 2011 American College of Rheumatology recommendations for the treatment of juvenile idiopathic arthritis: recommendations for the medical therapy of children with systemic juvenile idiopathic arthritis and tuberculosis screening among children receiving biologic medications. Arthritis Res Ther.

[3] Davies R, Southwood TR, Kearsley-Fleet L, Lunt M, Hyrich KL (2015). Medically significant infections are increased in patients with juvenile idiopathic arthritis treated with etanercept: results from the British Society for Paediatric and Adolescent Rheumatology Etanercept Cohort Study. Arthritis Rheumatol.

[4] Beukelman T, Haynes K, Curtis JR, Xie F, Chen L, Bemrich-Stolz CJ (2012). Rates of malignancy associated with juvenile idiopathic arthritis and its treatment. Arthritis Rheum.

[5] Tarkiainen M, Tynjala P, Vahasalo P, Lahdenne P (2015). Occurrence of adverse events in patients with JIA receiving biologic agents: long-term follow-up in a real-life setting. Rheumatology.

[6] Horneff G, Schmeling H, Biedermann T, Foeldvari I, Ganser G, Girschick HJ (2004). The German etanercept registry for treatment of juvenile idiopathic arthritis. Ann Rheum Dis.

[7] Horneff G, De Bock F, Foeldvari I, Girschick HJ, Michels H, Moebius D (2009). Safety and effectiveness of combination of etanercept and methotrexate compared to treatment with etanercept only in patients with juvenile idiopathic arthritis (JIA): preliminary data from the German JIA registry. Ann Rheum Dis.

[8] The European Network of Centres for Pharmacoepidemiology and Pharmacovigilance Data Source. http://www.encepp.eu/encepp/viewResource.htm?id=20591. Accessed 20 Mar 2020.

[9] Petty RE, Southwood TR, Manners P, Baum J, Glass DN, Goldenberg J (2004). International League of Associations for Rheumatology classification of juvenile idiopathic arthritis: second revision, Edmonton, 2001. J Rheumatol.

[10] Minden K, Niewerth M, Zink A, Seipelt E, Foeldvari I, Girschick H (2012). Long-term outcome of patients with JIA treated with etanercept, results of the biologic register JuMBO. Rheumatology (Oxford).

[11] Consolaro A, Ruperto N, Bazso A, Pistorio A, Magni-Manzoni S, Filocamo G (2009). Development and validation of a composite disease activity score for juvenile idiopathic arthritis. Arthritis Rheum.

[12] Giannini EH, Ruperto N, Ravelli A, Lovell DJ, Felson DT, Martini A (1997). Preliminary definition of improvement in juvenile arthritis. Arthritis Rheum.

[13] Wallace CA, Giannini EH, Huang B, Itert L, Ruperto N (2011). American College of Rheumatology provisional criteria for defining clinical inactive disease in select categories of juvenile idiopathic arthritis. Arthritis Care Res.

[14] Consolaro A, Bracciolini G, Ruperto N, Pistorio A, Magni-Manzoni S, Malattia C (2012). Remission, minimal disease activity, and acceptable symptom state in juvenile idiopathic arthritis: defining criteria based on the juvenile arthritis disease activity score. Arthritis Rheumatol..

[15] The International Council for Harmonization of Technical Requirements for Pharmaceuticals for Human Use. ICH harmonized guideline integrated addendum to ICH E6 (R1): guideline for good clinical practice E6 (R2). www.ich.org/fileadmin/Public_Web_Site/ICH_Products/Guidelines/Efficacy/E6/E6_R2__Step_4_2016_1109.pdf. Accessed 12 Dec 2019.

[16] Horneff G, Klein A, Oommen PT, Hospach A, Foeldvari I, Feddersen I (2016). Update on malignancies in children with juvenile idiopathic arthritis in the German BIKER registry. Clin Exp Rheumatol.

[17] Horneff G, Schulz AC, Klotsche J, Hospach A, Minden K, Foeldvari I (2017). Experience with etanercept, tocilizumab and interleukin-1 inhibitors in systemic onset juvenile idiopathic arthritis patients from the BIKER registry. Arthritis Res Ther.

[18] Lovell DJ, Reiff A, Ilowite NT, Wallace CA, Chon Y, Lin SL (2008). Safety and efficacy of up to eight years of continuous etanercept therapy in patients with juvenile rheumatoid arthritis. Arthritis Rheum.

[19] Zuber Z, Rutkowska-Sak L, Postepski J, Dobrzyniecka B, Opoka-Winiarska V, Kobusinska K (2011). ETN treatment in juvenile idiopathic arthritis: the Polish registry. Med Sci Monit.

[20] Prince FH, Twil M, Ten Cate R, van Rossum MA, Armbrust W, Hoppenreijs EP (2009). Long-term follow-up on effectiveness and safety of ETN in juvenile idiopathic arthritis: the Dutch national register. Ann Rheum Dis.

[21] Wallace CA, Ringold S, Bohnsack J, Spalding SJ, Brunner HI, Milojevic D (2014). Extension study of participants from the trial of early aggressive therapy in juvenile idiopathic arthritis. J Rheumatol.

[22] Beukelman T, Xie F, Chen L, Baddley JW, Delzell E, Grijalva CG (2012). Rates of hospitalized bacterial infections associated with juvenile idiopathic arthritis and its treatment. Arthritis Rheum.

[23] Foeldvari I, Constantin T, Vojinovic J, Horneff G, Chasnyk V, Dehoorne J (2019). Etanercept treatment for extended oligoarticular juvenile idiopathic arthritis, enthesitis-related arthritis, or psoriatic arthritis: 6-year efficacy and safety data from an open-label trial. Arthritis Res Ther.

[24] Giannini EH, Ilowite NT, Lovell DJ, Wallace CA, Rabinovich CE, Reiff A (2009). Long-term safety and effectiveness of etanercept in children with selected categories of juvenile idiopathic arthritis. Arthritis Rheum.

[25] Beukelman T, Xie F, Baddley JW, Chen L, Mannion ML, Saag KG (2016). The risk of hospitalized infection following initiation of biologic agents versus methotrexate in the treatment of juvenile idiopathic arthritis. Arthritis Res Ther.

[26] Barthel D, Ganser G, Kuester RM, Onken N, Minden K, Girschick HJ (2015). Inflammatory bowel disease in juvenile idiopathic arthritis patients treated with biologics. J Rheumatol.

[27] Sandborn WJ, Hanauer SB, Katz S, Safdi M, Wolf DG, Baerg RD (2001). Etanercept for active Crohn’s disease: a randomised, double-blind, placebo-controlled trial. Gastroenterology.

[28] Swart J, Giancane G, Horneff G, Magnusson B, Hofer M, Alexeeva E (2018). Pharmacovigilance in juvenile idiopathic arthritis patients treated with biologics or synthetic drugs: combined data of more than 15000 patients from Pharmachild and national registries. Arthritis Res Ther.

