# Supplementary material for: Long-term safety and effectiveness of etanercept in JIA: an 18-year experience from the BiKeR registry
Source: Arthritis Res Ther. 2020 Oct 29;22:258. doi: 10.1186/s13075-020-02326-5 (PMC7597050; doi:10.1186/s13075-020-02326-5)
Supplement: Supplementary file 1 [file 13075_2020_2326_MOESM1_ESM.docx]

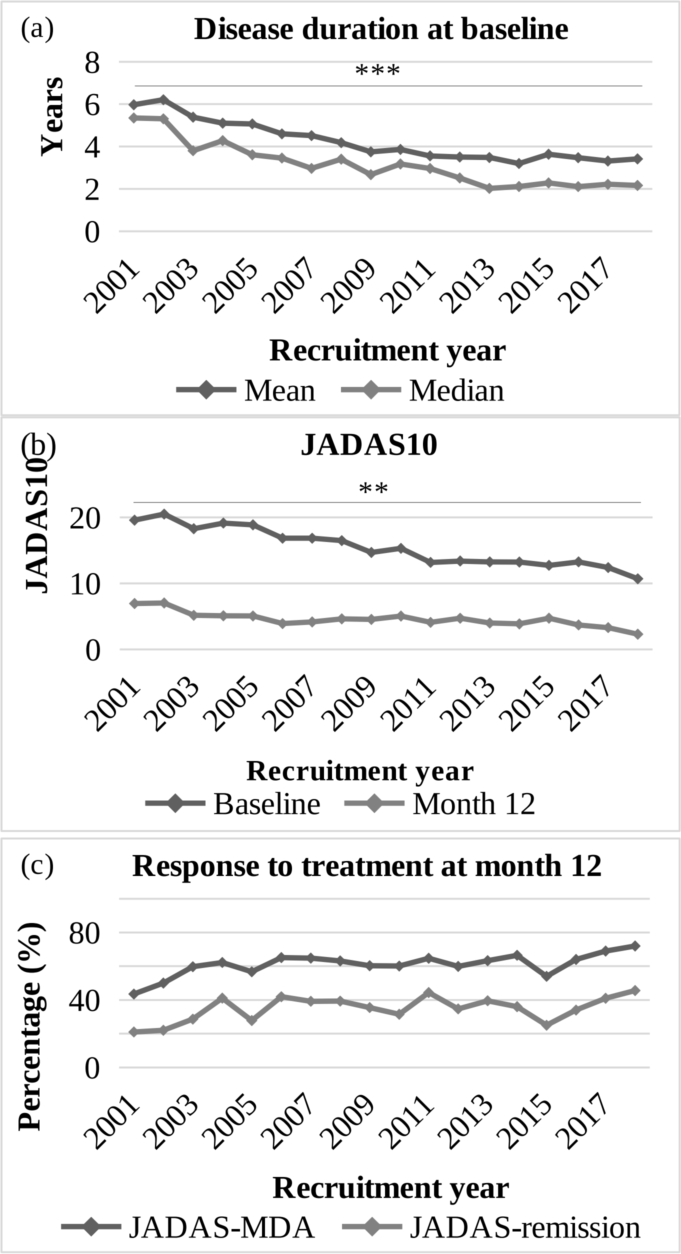
(a)
Disease duration at baseline
Years
***
8
6
4
2
0
2001
2003
2005
2007
2009
2011
2013
2015
2017
Recruitment year
Mean
Median
(b)
JADAS10
JADAS10
**
20
10
0
2001
2003
2005
2007
2009
2011
2013
2015
2017
Recruitment year
Baseline
Month 12
(c)
Response to treatment at month 12
Percentage (%)
80
40
0
2001
2003
2005
2007
2009
2011
2013
2015
2017
Recruitment year
JADAS-MDA
JADAS-remission
