# Supplementary material for: Long-term safety and effectiveness of etanercept in JIA: an 18-year experience from the BiKeR registry
Source: Arthritis Res Ther. 2020 Oct 29;22:258. doi: 10.1186/s13075-020-02326-5 (PMC7597050; doi:10.1186/s13075-020-02326-5)
Supplement: Supplementary file 2 [file 13075_2020_2326_MOESM2_ESM.docx]

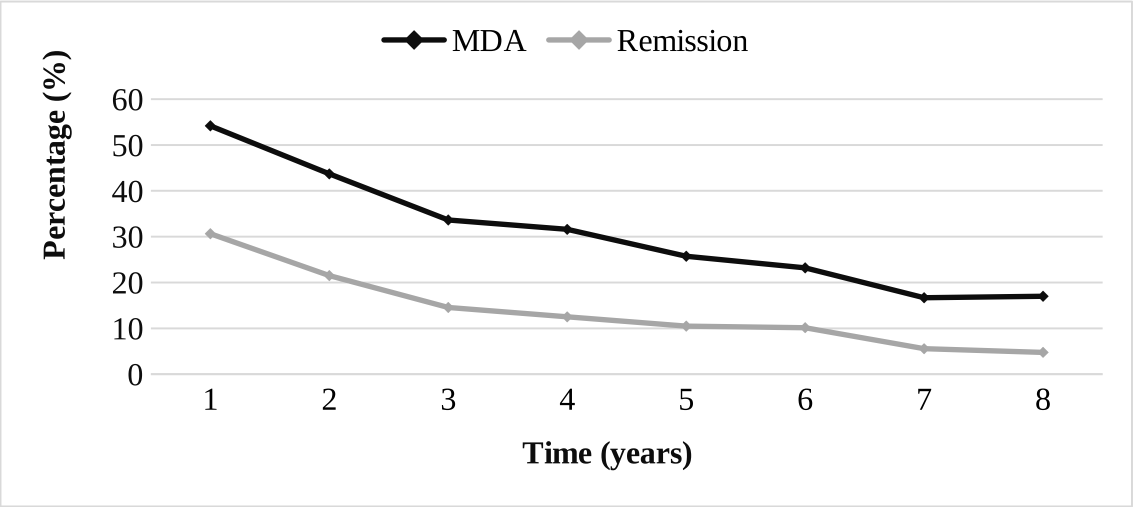

MDA
Remission
Percentage (%)
60
50
40
30
20
10
0
1
2
3
4
5
6
7
8
Time (years)
